# Evaluation of a National Quality Improvement Collaborative for Improving Cancer Screening

**DOI:** 10.1001/jamanetworkopen.2022.42354

**Published:** 2022-11-16

**Authors:** Rachel Hae-Soo Joung, Timothy W. Mullett, Scott H. Kurtzman, Sarah Shafir, James B. Harris, Katharine A. Yao, Karl Y. Bilimoria, William G. Cance, Heidi Nelson

**Affiliations:** 1 Northwestern University Feinberg School of Medicine, Department of Surgery, Chicago, Illinois; 2 American College of Surgeons Cancer Programs, Chicago, Illinois; 3 Markey Cancer Center, University of Kentucky, Lexington; 4 National Accreditation Program for Breast Centers, Chicago, Illinois; 5 Waterbury Hospital, Waterbury, Connecticut; 6 American Cancer Society, Atlanta, Georgia; 7 University of Nevada Reno School of Medicine, Reno; 8 NorthShore University Health System, Evanston, Illinois; 9 Now with GRAIL, LLC

## Abstract

**Importance:**

Cancer screening deficits during the first year of the COVID-19 pandemic were found to persist into 2021. Cancer-related deaths over the next decade are projected to increase if these deficits are not addressed.

**Objective:**

To assess whether participation in a nationwide quality improvement (QI) collaborative, Return-to-Screening, was associated with restoration of cancer screening.

**Design, Setting, and Participants:**

Accredited cancer programs electively enrolled in this QI study. Project-specific targets were established on the basis of differences in mean monthly screening test volumes (MTVs) between representative prepandemic (September 2019 and January 2020) and pandemic (September 2020 and January 2021) periods to restore prepandemic volumes and achieve a minimum of 10% increase in MTV. Local QI teams implemented evidence-based screening interventions from June to November 2021 (intervention period), iteratively adjusting interventions according to their MTVs and target. Interrupted time series analyses was used to identify the intervention effect. Data analysis was performed from January to April 2022.

**Exposures:**

Collaborative QI support included provision of a Return-to-Screening plan-do-study-act protocol, evidence-based screening interventions, QI education, programmatic coordination, and calculation of screening deficits and targets.

**Main Outcomes and Measures:**

The primary outcome was the proportion of QI projects reaching target MTV and counterfactual differences in the aggregate number of screening tests across time periods.

**Results:**

Of 859 cancer screening QI projects (452 for breast cancer, 134 for colorectal cancer, 244 for lung cancer, and 29 for cervical cancer) conducted by 786 accredited cancer programs, 676 projects (79%) reached their target MTV. There were no hospital characteristics associated with increased likelihood of reaching target MTV except for disease site (lung vs breast, odds ratio, 2.8; 95% CI, 1.7 to 4.7). During the preintervention period (April to May 2021), there was a decrease in the mean MTV (slope, −13.1 tests per month; 95% CI, −23.1 to −3.2 tests per month). Interventions were associated with a significant immediate (slope, 101.0 tests per month; 95% CI, 49.1 to 153.0 tests per month) and sustained (slope, 36.3 tests per month; 95% CI, 5.3 to 67.3 tests per month) increase in MTVs relative to the preintervention trends. Additional screening tests were performed during the intervention period compared with the prepandemic period (170 748 tests), the pandemic period (210 450 tests), and the preintervention period (722 427 tests).

**Conclusions and Relevance:**

In this QI study, participation in a national Return-to-Screening collaborative with a multifaceted QI intervention was associated with improvements in cancer screening. Future collaborative QI endeavors leveraging accreditation infrastructure may help address other gaps in cancer care.

## Key Points

**Question:**

Is participation in a nationwide quality improvement (QI) collaborative, Return-to-Screening, associated with restoration of deficits in cancer screening during the COVID-19 pandemic?

**Findings:**

In this study, 859 QI projects to improve cancer screening were completed by 786 accredited cancer programs, with the majority (79%) accomplishing the goal of restoring prepandemic volumes and achieving a minimum of 10% growth in screening.

**Meaning:**

These findings suggest that the Return-to-Screening national collaborative effort was associated with improvements in cancer screening, and such QI endeavors leveraging accreditation infrastructure can be a model for addressing other gaps in cancer care.

## Teaser

This quality improvement study assesses whether participation in a nationwide quality improvement collaborative, Return-to-Screening, was associated with restoration of cancer screening during the COVID-19 pandemic.

## Introduction

The early months of the COVID-19 pandemic were associated with an abrupt cessation in cancer screening, with more than 9 million screening tests estimated to have been missed across the US in 2020.^1^ It is anticipated that these deficits will lead to unnecessary cancer-related morbidity and mortality.^2,3^ In addition, the persistence of the pandemic, with ongoing uncertainty among patients, and resource constraints within the health care system, have led to concerns that there may be perpetual setbacks in cancer screening.

In early 2021, the American College of Surgeons (ACoS) Cancer Programs and the American Cancer Society (ACS) developed a Return-to-Screening national quality improvement (QI) collaborative to help Commission on Cancer (CoC) and National Accreditation Program of Breast Centers (NAPBC) accredited programs estimate local screening deficits, restore screening to prepandemic volumes, and work toward addressing the backlog created by the missed screenings.^4,5^ Although other reports^6,7,8^ have described efforts to mitigate the negative impact of the pandemic on cancer screening, most of these studies were single-institution experiences or modeling studies based on estimated effects. To our knowledge, the present study is the largest QI collaborative to address the pandemic-related deficits in cancer screening. The objective of this report is to assess whether participation in the nationwide Return-to-Screening QI collaborative was associated with restoration of cancer screening to prepandemic levels.

## Methods

This QI study used an interrupted time series analyses, with each QI project at the facility level serving as its own unit of reference. Standards for Quality Improvement Reporting Excellence (SQUIRE) reporting guidelines version 2.0 were referenced as a framework for quality improvement methods, data analysis, and reporting.^9^ This study was determined to be exempt from institutional review board oversight and the need for informed consent given the absence of patient-level identifiers, in accordance with 45 CFR §46.

### Study Population and Enrollment in the Return-to-Screening Collaborative

Enrollment for this effort was open from April 8, 2021, through June 1, 2021, for all 1456 CoC programs that treat nearly 70% of US patients with recently diagnosed cancer annually, and 582 NAPBC programs that provide high-quality comprehensive breast cancer services.^10^ These accredited programs include hospitals, treatment centers, and other cancer facilities that demonstrate ongoing compliance with a rigorous set of standards designed to support the full continuum of quality cancer care.^11^ CoC-accredited programs are further categorized according to the type of facility, program structure, services provided, and yearly patient case volume.^4,12,13^

Participation in the Return-to-Screening Collaborative and successful completion of each QI project led to program accruals of existing relevant standards in cancer screening event, QI initiative, and clinical research. CoC programs were enrolled in 1 or more independent QI projects in breast, colon, lung, and cervical cancer screening, whereas NAPBC programs were enrolled in breast cancer screening projects, and multidisciplinary local QI teams involving key stakeholders were assembled. Teams submitted program-level data on disease-specific monthly screening test volumes (MTVs), a count of the number of screening tests performed each month. Each team specified which screening tests were measured from a list of recommended screening tests for each disease site (eTable 1 in Supplement 1).^14,15,16,17^ Prepandemic and pandemic MTVs were calculated using the mean of the number of screening tests performed in September 2019 and January 2020, and in September 2020 and January 2021, respectively. Monthly screening deficits were calculated according to the prepandemic and pandemic MTV, as described in our previous report,^4^ and targets were set to increase MTV by a minimum of 10%. Furthermore, programs had to achieve 1 of 2 goals depending on their baseline screening deficit: either restore MTV to prepandemic volumes or exceed prepandemic volumes to address the backlog of missed cancer screenings.^18^

### Return-to-Screening Coordinating Committee

The Return-to-Screening coordinating committee, composed of executive committee members of the ACoS, CoC, NAPBC, and ACS, met regularly throughout the study to develop the QI protocol and educational webinar materials, review data, and promote collaborative learning. The model for improvement was used to coach all participating local QI teams on the plan-do-study-act (PDSA) cycles.^19^ Bimonthly webinars on QI, implementation, and screening interventions were attended by local QI team members. Teaching on data use and analysis was provided to help participants with performance monitoring using their site-specific, real-time data. Aggregated midterm data were presented halfway into the study. Select programs that were able to attain target monthly goals early were identified and invited as speakers during disease-specific focus groups to highlight attributes leading to early successes. QI experts facilitated discussion in focus groups to share challenges faced by local teams and discuss potential solutions to address barriers.

### Screening Interventions

From June 2021 to November 2021, local QI teams implemented 1 or more evidence-based interventions on improving cancer screening recommended by the Community Preventive Services Task Force and in keeping with local needs and environment.^20^ Interventions were grouped into 3 strategies: interventions to increase patient demand, increase delivery of screening services, or increase community access (eTable 2 in Supplement 1). At the end of each month, teams were instructed to evaluate their monthly screening volume against their target and assess the need to adjust screening interventions. Data were prospectively recorded in real time in REDCap, which helped local teams with iterative assessments of MTVs and the type and number of interventions implemented each month. Weekly updates were posted in the ACoS Cancer Program’s newsletter, and monthly emails were sent with reminders and instructions on data collection and performance monitoring.

### Outcome Variables

The primary outcome was the rate of programs reaching their target MTV during the intervention period. Covariates included program location, type of institution, and baseline screening deficit, classified as none, 0 to 10%, and more than 10%. Programs were categorized as having conducted a multicomponent intervention strategy if they consistently conducted 2 or more strategies.

### Statistical Analysis

The numbers of monthly screening tests across all participating programs were summed to estimate the aggregate number of screening tests for 4 representative periods: prepandemic (mean monthly volumes in September 2019 and January 2020), pandemic (mean monthly volumes in September 2020 and January 2021), preintervention baseline (April and May 2021), and intervention (June through November 2021). Prepandemic and pandemic monthly aggregates were multiplied by 6 to estimate 6-month cumulative sums; similarly, for the preintervention cumulative estimate, April and May monthly aggregates were added and multiplied by 3. Monthly aggregates were summed from June to November to calculate the cumulative number of screening tests performed during the intervention period. We then used these to calculate counterfactual differences in the number of screening tests.

Bivariate associations between reaching target and covariates were examined using χ^2^ tests for categorical variables. Multivariable logistic regression models were estimated to evaluate associations between baseline screening deficits, interventions, and outcomes, adjusting for program characteristics. Interrupted time series analyses accounting for autocorrelation were used to estimate immediate and trend changes in MTVs before and after implementation of screening interventions.^21,22^ Statistical significance was set at *P* < .05, and all tests were 2-tailed. All analyses were performed using STATA MP statistical software version 17.0 (StataCorp). Data analysis was performed from January to April 2022.

## Results

### Characteristics of Programs Participating in Return-to-Screening Collaborative

Overall, 786 programs (528 CoC and 258 NAPBC) electively enrolled in the Return-to-Screening national QI collaborative, resulting in a 36.3% (528 of 1456 programs) participation rate from all CoC-accredited programs and 44.3% (258 of 582 programs) participation rate from all NAPBC-accredited programs. All 258 NAPBC programs completed projects for breast cancer screening. Of 528 CoC programs, 468 completed projects for 1 disease site, 50 for 2 disease sites, 7 for 3 disease sites, and 3 for all 4 disease sites, resulting in 859 independent QI projects (452 for breast cancer, 134 colorectal cancer, 244 for lung cancer, and 29 for cervical cancer).

Project-specific baseline screening deficits are illustrated in Table 1. Of 859 disease-specific local QI projects, 32% (276 projects) had a baseline screening deficit of more than 10%, ranging from 22% of breast projects to 61% of colorectal projects. However, more than one-half of the programs (136 of 244 programs [56%]) conducting projects in lung cancer screening did not have screening deficits at baseline before starting interventions.

**Table 1.** Characteristics of Cancer Programs Participating in the National Return-to-Screening Quality Improvement Study

Characteristic	Programs, No. (%)			
	All projects	Stratified cohort according to baseline screening deficit		
		Screening deficit		No screening deficit
		≥10%	<10%	
Total	859 (100)	276 (32)	206 (24)	377 (44)
Disease site				
Breast	452 (53)	101 (22)	149 (33)	202 (45)
Colon	134 (16)	81 (61)	23 (17)	30 (22)
Lung	244 (28)	82 (34)	26 (11)	136 (56)
Cervix	29 (3)	12 (41)	8 (28)	9 (31)
Facility location				
Northeast	177 (21)	64 (36)	35 (20)	78 (44)
Midwest	224 (26)	54 (24)	55 (25)	115 (51)
South	317 (37)	105 (33)	78 (25)	134 (42)
West	139 (16)	53 (38)	38 (27)	48 (35)
Type of institution				
Community cancer program	134 (16)	49 (37)	31 (23)	54 (40)
Comprehensive community cancer program	237 (28)	90 (38)	42 (18)	105 (44)
Academic or research program	73 (9)	25 (34)	24 (33)	24 (33)
Integrated network cancer program	131 (15)	50 (38)	27 (21)	54 (41)
Other program	26 (3)	12 (46)	5 (19)	9 (35)
National Accreditation Program of Breast Centers–accredited breast center	258 (30)	50 (19)	77 (30)	131 (51)

### Screening Interventions

Overall, monthly adherence to intervention implementation ranged from 91% to 95%. Patient reminders were the most common screening intervention (67%-72%), followed by media (43%-62%) and dissemination of guidelines and messaging to patients (43%-61%) (eTable 3 in Supplement 1). The median (IQR) number of interventions implemented by programs per month was 4 (2-6) for all months except in October 2021 (median [IQR], 5 [3-8] interventions). The eFigure in Supplement 1 demonstrates the proportion of programs that implemented various intervention strategies each month. A total of 789 programs (92%) implemented more than 1 type of intervention strategies to target more than 1 levels (ie, patient, physician, and community).

### Outcomes

Overall, 676 programs (79%) were able to reach their target MTV as set on the basis of their baseline screening deficits (Table 2). On bivariate analysis, there were significant differences in reaching target according to the disease site, with only 59% of cervical cancer projects reaching the target MTV vs 75% in breast cancer, 80% in colorectal cancer, and 87% in lung cancer. Multivariable modeling including location, type of institution, baseline screening deficits, and intervention approach demonstrated that disease site was the only factor associated with reaching target. Projects on lung cancer screening were almost 3 times as likely to have reached the target goal compared with projects on breast cancer screening (odds ratio, 2.8; 95% CI, 1.7-4.7; *P* < .001), and projects on colorectal cancer screening were 1.8 times more likely (95% CI, 1.0-3.2; *P* = .04) to do so. There were no differences in reaching target goal between programs that conducted multicomponent intervention strategy or single component strategy.

**Table 2.** Program Factors Associated With Reaching Target Monthly Screening Test Volumes After Participation in the National Return-to-Screening Quality Improvement Study^a^

Characteristic	Programs, No. (%)		Adjusted OR (95% CI)	*P* value
	Reached target			
	No (n = 181 [21%])	Yes (n = 676 [79%])		
Disease site				
Breast	112 (25)	340 (75)	1 [Reference]	NA
Colon	27 (20)	107 (80)	1.8 (1.0-3.2)	.04
Lung	31 (13)	213 (87)	2.8 (1.7-4.7)	<.001
Cervix	12 (41)	17 (59)	0.6 (0.3-1.3)	.18
Facility location				
Northeast	36 (20)	141 (80)	1 [Reference]	NA
Midwest	51 (23)	173 (77)	1.0 (0.6-1.6)	.88
South	60 (19)	257 (81)	1.2 (0.8-2.0)	.38
West	34 (25)	105 (76)	0.9 (0.5-1.6)	.80
Type of institution				
Community cancer program	27 (20)	107 (80)	1 [Reference]	NA
Comprehensive community cancer program	44 (19)	193 (81)	1.0 (0.6-1.8)	.98
Academic or research program	15 (21)	58 (80)	0.9 (0.4-1.9)	.78
Integrated network cancer program	30 (23)	101 (77)	0.7 (0.4-1.3)	.23
Other program	6 (23)	20 (77)	0.8 (0.3-2.3)	.70
National Accreditation Program of Breast Centers–accredited breast center	60 (23)	198 (77)	1.2 (0.7-2.0)	.62
Baseline screening deficit				
None	73 (19)	304 (81)	1 [Reference]	NA
0%-10%	45 (22)	161 (78)	1.1 (0.7-1.6)	.82
>10%	64 (23)	212 (77)	0.8 (0.5-1.2)	.21
Intervention strategy				
Single component	12 (17)	58 (83)	1 [Reference]	NA
Multicomponent	170 (22)	619 (79)	0.8 (0.4-1.6)	.51

Abbreviations: NA, not applicable; OR, odds ratio.

^a^ Target monthly screening test volume for each quality improvement project was calculated before the intervention according to differences in mean monthly screening test volume between representative prepandemic and pandemic time period. Reaching target during the 6-month intervention period indicated that programs achieved a minimum of 10% increase in monthly screening test volume as well as close any residual gaps in screening.

**Figure 1.**
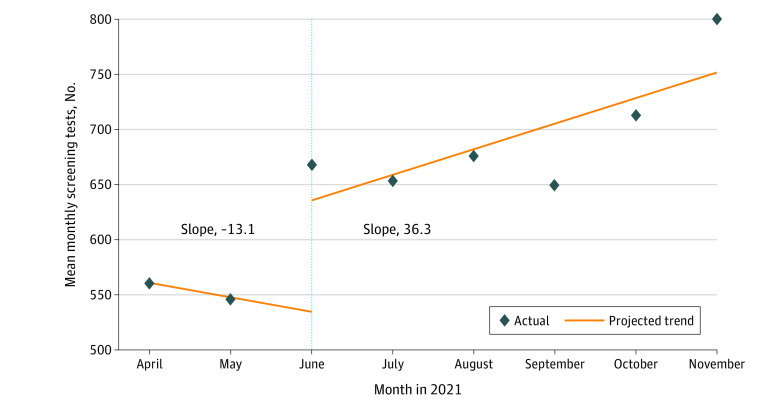
Mean Monthly Screening Test Volumes in April Through November 2021 April and May 2021 is the preintervention period. Vertical dotted line indicates the start of the intervention period (June 2021).

Figure 1 demonstrates the trends in monthly screening test volumes from April through November 2021. During the preintervention period, there was a decrease in mean MTVs (slope, −13.1 tests per month; 95% CI, −23.1 to −3.2 tests per month). With the start of interventions, there was a significant increase in MTV (slope, 101.0 tests per month; 95% CI, 49.1 to 153.0 tests per month; *P* < .001), followed by a statistically significant increase in the monthly trend compared with the preintervention trend, with the mean MTV increasing by 36.3 tests per month (95% CI, 5.3 to 67.3 tests per month; *P* = .02). Disease-specific interrupted time-series analyses are summarized in eTable 4 in Supplement 1.

**Figure 2.**
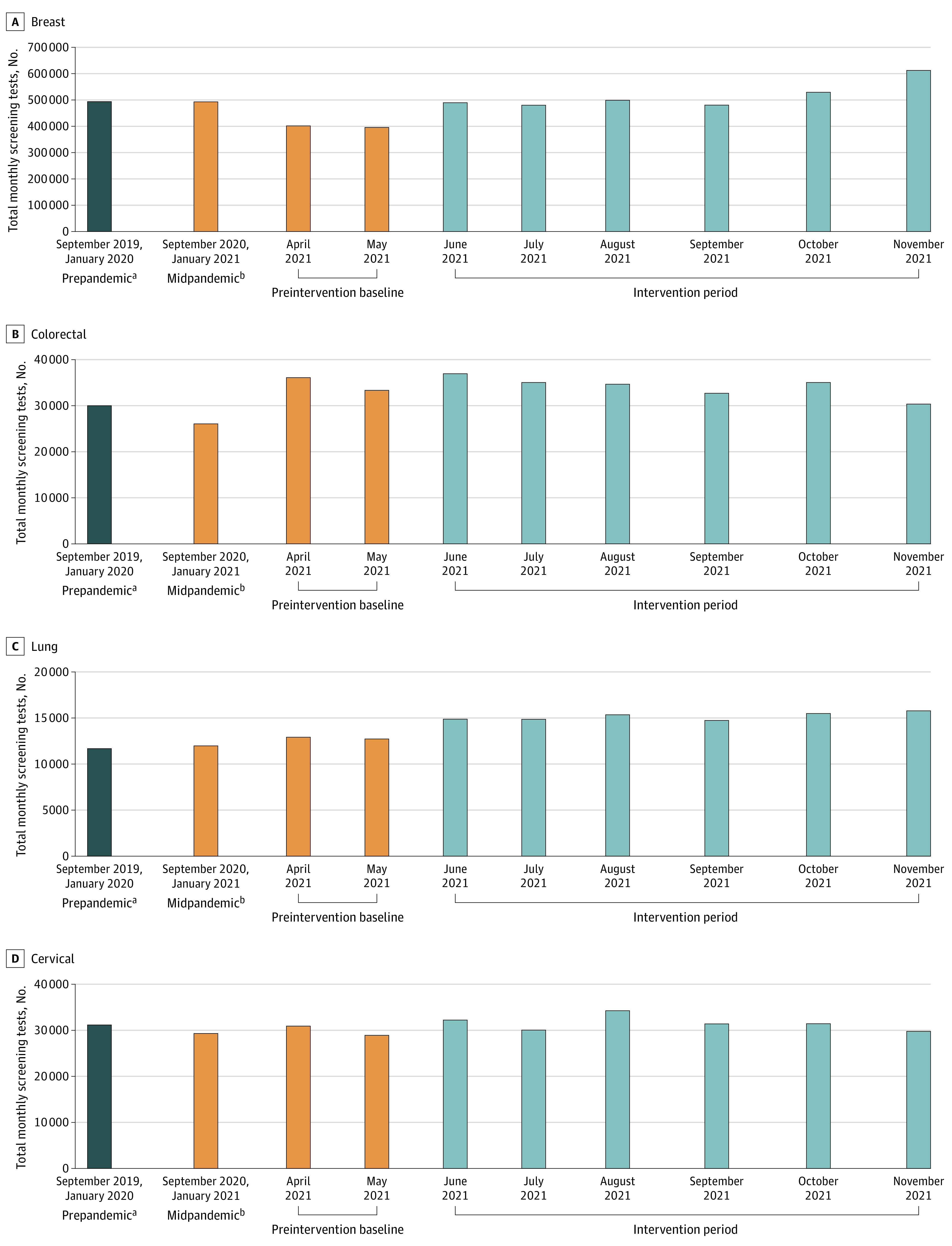
Total Number of Monthly Screening Tests in Breast, Colorectal, Lung, and Cervical Cancers Across Time ^a^The prepandemic period data are shown as monthly mean volumes from September 2019 and January 2020. ^b^The early pandemic period data are shown as monthly mean volumes for September 2020 and January 2021.

Figure 2 displays the total number of monthly screening tests in breast, colorectal, lung, and cervical cancer screening across all participating programs. The total number of monthly screening tests across all participating programs and counterfactual differences across periods are shown in Table 3. During the intervention period, additional screening tests were performed compared with the prepandemic period (170 748 tests), the pandemic period (210 450 tests), and the preintervention period (722 427 tests). Programs that completed breast QI projects conducted 122 404 additional screening tests during the intervention period compared with the prepandemic period, and an estimated 702 145 additional screening tests compared with the preintervention baseline period.

**Table 3.** Total Number of Monthly Screening Tests Across All Participating Programs

Variable	Prepandemic, September 2019 and January 2020^a^	Pandemic, September 2020 and January 2021^a^	Preintervention baseline		Intervention period					
			April 2021	May 2021	June 2021	July 2021	August 2021	September 2021	October 2021	November 2021
Disease site										
Breast	494 016	492 807	400 982	393 803	489 451	481 048	496 127	478 788	529 956	611 130
Colon	29 984	26 094	36 158	33 342	37 036	35 043	34 797	32 727	35 126	30 381
Lung	11 687	11 960	12 936	12 748	14 903	14 881	15 366	14 772	15 499	15 860
Cervix	31 210	29 419	30 985	28 947	32 208	29 942	34 323	31 426	31 523	29 817
Cumulative total^b^	566 897	560 280	481 061	468 840	573 598	560 914	580 613	557 713	612 104	687 188
6-mo Estimate^c^	3 401 382	3 361 680	2 849 703		3 572 130					
Counterfactual difference (vs intervention period)^d^	170 748	210 450	722 427		NA					

Abbreviation: NA, not applicable.

^a^ Prepandemic and pandemic values are means.

^b^ Refers to cumulative total number of monthly screening tests across all disease sites and projects.

^c^ The 6-month estimate for prepandemic and pandemic period was calculated by multiplying mean monthly cumulative total by 6. For the preintervention 6-month estimate, total number of screening tests from April and May were added and multiplied by 3. For the intervention 6-month period, total number of screening tests from June to November were summed.

^d^ Counterfactual differences were calculated for prepandemic, pandemic, and preintervention baseline period compared with the intervention period.

## Discussion

This Return-to-Screening national QI collaborative was originally launched with the intent of addressing missed screenings associated with the onset of the pandemic in early 2020. As we enrolled accredited programs and set monthly screening targets for local QI project implementation in this quality improvement study, we discovered that more than one-half of our participating programs had still not returned to prepandemic levels of monthly screening well into 2020 and early 2021.^4^ In fact, overall monthly screening volumes in April and May 2021 were lower than those used to set targets in September 2020 and January 2021. Despite these ongoing declines in screening, 786 accredited programs completed 859 coordinated QI projects and 79% reached their target. Our analyses confirmed a significant and sustained increase in screening over the intervention period, resulting in 722 427 counterfactual additional screenings when compared with the immediate preintervention period.

The fact that most of the participating accredited programs were able to meet their screening targets supports the validity of establishing QI collaboratives within our accreditation programs to address major problems in cancer care. Furthermore, we did not find that the success of programs in reaching targets was contingent on or adversely affected by geography, type of institution, or baseline screening deficit. We anticipated, in advance of conducting the study, that these factors might play a role in the success of programs reaching targets because of the uneven distribution of pandemic surges across geography and the secondary impact of surges on health care resources. For example, we were concerned that programs with larger baseline screening deficits would be disproportionately disadvantaged and unlikely to reverse the downward trends and reach targets because of potentially limited health care resources. Similarly, we imagined it would be difficult for programs to grow screening volumes by 10%, exceeding prepandemic volumes, as was the target for those programs with minimal or no screening deficits. The fact that most programs, irrespective of their differences, were able to meet their goals supports that the use of a national PDSA protocol with local tailored approach can be successful across a wide range of accredited programs and environments.

Although program and environmental factors were not associated with reaching targets, we did identify variable outcomes depending on the disease site. An unexplainable and surprising finding was the notable decline in monthly screening volumes for breast cancer immediately before the intervention period. Even with these ongoing challenges, programs that completed breast QI projects conducted 122 404 additional screening tests during the intervention period compared with prepandemic period, and an estimated 702 145 additional screening tests compared with the preintervention baseline period, leading to the largest contribution of the overall impact of this national QI effort on cancer screening.

With regard to the other disease sites, we observed some inconsistent results that may relate to general differences in screening. First, even though the overall aggregate volumes for colon cancer screening were not comparable to breast cancer screening, projects on colorectal cancer screening were 1.8 times more likely to reach their target compared with projects on breast screening. This was unexpected given that we and others have previously reported that colorectal cancer screening was not only slower to recover after the initial impact of the pandemic, but in fact had not completely recovered even as late as September 2020 and January 2021.^1,4^ To our surprise, colorectal screening volumes increased substantially in April and May 2021 and consistently surpassed prepandemic volumes for the remainder of the intervention period. We can speculate that some of the additional screens may have been due to the increased use of alternative home-based screening tests, which were promoted during the pandemic.^23,24^

Similarly, lung cancer screening projects were more likely to reach target compared with breast projects. What is unique about lung cancer screening is that the screening volumes were relatively unaffected by the pandemic. We noted that baseline monthly screenings during the pandemic were higher than before the pandemic, consistent with what others have reported,^25^ and they continued to increase after the interventions were implemented. The minimal nationwide declines in lung cancer screening may be explained by its relatively less invasive form of screening. Additionally, it is one of the most underused screenings; thus, there may not have been much room for further reductions.^26,27^

Despite some variability in outcomes across disease sites, we consider this effort to be a success, which we attribute to 3 key factors: the general motivation of health care professionals to reverse pandemic-related screening deficits, the presence of existing cancer accreditation infrastructure, and the rapid formation of a national cancer QI collaborative during a time when our health care system was in crisis. In fact, it was the crisis in cancer care that led to the creation of what we believe to be the nation’s largest cancer QI collaborative. This would not have been possible without the long-standing relationship between ACoS and ACS, the deep-rooted national infrastructure of the CoC and NAPBC, and the profound commitment and leadership of these professional organizations to improving cancer care. Together, these groups in the form of a coordinating committee were able to leverage existing toolkits and expertise in QI implementation and cancer screening to produce and disseminate a readily accessible national PDSA protocol. In addition, the coordinating committee provided central support for enrollment, data management, QI education, communications, and peer-to-peer shared learning on interventions, all of which are important elements to bring about meaningful QI, as has been well described by others.^28^ Finally, the CoC and NAPBC leadership were able to adapt existing standards in screening, QI, and clinical research to meet the immediate needs of the Return-to-Screening effort and establish new compliance requirements for individual programs.

The program-level CoC and NAPBC infrastructure was similarly contributory to the success of this effort. First, participating programs were familiar and experienced with screening, QI, and clinical research because of the existing standards. Second, all participating CoC-accredited and NAPBC-accredited programs had well-established and structured cancer committees complete with leadership, administration, multidisciplinary teams, and a culture of teamwork. Although it is clear to us that cancer accreditation played a large role in the success of this effort, the reverse is also true, that without the overarching QI collaborative, individual efforts by accredited programs would likely not have been as successful.^29^ This national QI collaborative reinforced the importance of QI among our programs and provided structured education on QI methods, all of which have implications for the future conduct of cancer QI.

### Limitations

This study has limitations that should be addressed. We have to acknowledge that this effort was not a rigorously controlled study; rather, it was a rapid response to reverse the adverse impact of the pandemic on cancer screening. As such, there was limited ability to include nonparticipant controls and account for confounders, especially related to fluctuations in the pandemic, which we adjusted for using a pre-post design and interrupted time series analyses. Next, we did not include patient-level demographic data, making a broad and highly theoretical assumption that the patient populations eligible for and receiving screening remained relatively constant across institutions throughout the study period. In addition, the fact that accredited programs self-selected participation and projects does limit the potential generalizability, which is somewhat overcome by the high participation across diverse types of institutions. Furthermore, despite a relatively short intervention period, we found a high success rate in programs achieving their goals and anticipate that we would have seen even greater response with a longer study duration.

## Conclusions

The 859 QI projects conducted by accredited cancer programs as part of the Return-to-Screening national QI collaborative were associated with a reversal of screening deficits associated with the COVID-19 pandemic and resulted in a significant additional number of screening tests. The success in achieving target goals by the majority of participating programs, irrespective of their differences, suggests that national QI collaboratives may be an effective large-scale approach to improving cancer care.

## Notes

Supplement 1. **eTable 1.** Screening Tests Selected for Projects **eTable 2.** Screening Interventions **eTable 3.** Interventions Implemented by Month **eTable 4.** Coefficients From Interrupted Time Series Models, Stratified by Disease Site **eFigure.** Intervention Strategies Implemented During the Intervention Months (June to November)

Supplement 2.
Return-to-Screening Quality Improvement Collaborative Group Members
