# Supplementary material for: Evaluation of a National Quality Improvement Collaborative for Improving Cancer Screening
Source: JAMA Netw Open. 2022 Nov 16;5(11):e2242354. doi: 10.1001/jamanetworkopen.2022.42354 (PMC9669819; doi:10.1001/jamanetworkopen.2022.42354)
Supplement: Supplement 1. — eTable 1. Screening Tests Selected for Projects eTable 2. Screening Interventions eTable 3. Interventions Implemented by Month eTable 4. Coefficients From Interrupted Time Series Models, Stratified by Disease Site eFigure. Intervention Strategies Implemented During the Intervention Months (June to November) [file jamanetwopen-e2242354-s001.pdf]

Supplemental Online Content

Joung RHS, Mullett TW, Kurtzman SH, et al; Return-to-Screening Quality Improvement Collaborative. Evaluation of a national quality improvement collaborative for improving cancer screening. *JAMA Netw Open*. 2022;5(11):e2242354. doi:10.1001/jamanetworkopen.2022.42354

eTable 1. Screening Tests Selected for Projects
eTable 2. Screening Interventions
eTable 3. Interventions Implemented by Month
eTable 4. Coefficients From Interrupted Time Series Models, Stratified by Disease Site
eFigure. Intervention Strategies Implemented During the Intervention Months (June to November)

This supplemental material has been provided by the authors to give readers additional information about their work.

© 2022 Joung RHS et al. *JAMA Network Open.*

eTable 1. Screening Tests Selected for Projects

Disease Site	Measured Screening Tests	No. (%)
Breast	Screening Mammography only	414 (91.6)
	Combination (Mammography, MRI)	38 (8.4)
Colon	Colonoscopy only	86 (64.2)
	Stool-based only	4 (3.0)
	Combination	44 (32.8)
Lung	Low-dose CT scan	224 (100.0)
Cervix	Pap Smear only	17 (58.6)
	Pap Smear + Human Papillomavirus Test	12 (41.4)

© 2022 Joung RHS et al. *JAMA Network Open.*

eTable 2. Screening Interventions

**Strategy**	**Intervention**	**Sub-category / examples**
Increase Patient Demand	Patient Reminders	Patient outreach by healthcare providers to eligible and at-risk patients (eg, phone calls, EMR portal, email, text messages, letters)
		Facility/Institution-level outreach (eg, automated notifications to eligible patients within health system)
	Patient Education	One-on-one education (ie, deliver information to individuals about indications for, benefits of, and ways to obtain cancer screening)
		Group education (ie, group education is usually conducted by health professionals or by trained lay people who use presentations or other teaching aids in a lecture or interactive format to a variety of groups)
	Dissemination of guideline/messaging	Dissemination of guidelines and messaging information to patients across the hospital system (eg, banners/posters, pamphlets, hospital website)
		Dissemination of guidelines and messaging information across community sites (eg, vaccination sites, grocery stores, pharmacies, etc.)
	Media	Institution social media posts and/or press releases (eg, Twitter, Facebook)
		Collaboration with local TV/radio/news channels to communicate the importance of cancer screening and the safety of screening during the COVID-19 pandemic
Increase Provider Delivery	Provider Awareness & Education	Dissemination of guidelines and messaging information to primary care practitioners (as defined by institution)
		Dissemination of guidelines and messaging information to specialists (as defined by institution)
	Provider Reminder & Recall	Reminders sent to health care providers that it is time for a patient's cancer screening test or that the patient is overdue for screening
	Provider Assessment & Feedback	Interventions aimed at evaluating provider performance in delivering or offering screening to patients
Increase Community Access	Reduce Socioeconomic Barriers	Eg, collaboration with local community group leaders to reach underserved populations at risk for screening disparities
	Reduce Structural Barriers	Eg, modifying hours of service
	Reduce Economic Barriers	Eg, reduce out-of-pocket costs

© 2022 Joung RHS et al. *JAMA Network Open.*

eTable 3. Interventions Implemented by Month

Interventions	June	July	August	Sept	October	Nov
	No. (%)					
**Increase Patient Demand**	**768 (89.4)**	**755 (87.9)**	**754 (87.8)**	**762 (88.7)**	**787 (91.6)**	**744 (86.6)**
Patient Reminders	602 (70.1)	605 (70.4)	602 (70.1)	582 (67.8)	618 (71.9)	579 (67.4)
Patient Education	292 (34.0)	302 (35.2)	290 (33.8)	298 (34.7)	372 (43.3)	293 (34.1)
Dissemination of Guideline/Messaging	373 (43.4)	399 (46.4)	412 (48.0)	437 (50.9)	527 (61.4)	421 (49.0)
Media	389 (45.3)	371 (43.2)	424 (49.4)	452 (52.6)	528 (61.5)	407 (47.4)
**Increase Provider Delivery**	**503 (58.6)**	**494 (57.5)**	**521 (60.7)**	**495 (57.6)**	**524 (61.0)**	**472 (54.9)**
Provider Awareness & Education	367 (42.7)	345 (40.2)	375 (43.7)	365 (42.5)	403 (46.9)	342 (39.8)
Provider Reminder & Recall	259 (30.2)	267 (31.1)	270 (31.4)	272 (31.7)	272 (31.7)	252 (29.3)
Provider Assessment & Feedback	117 (13.6)	124 (14.4)	129 (15.0)	134 (15.6)	131 (15.3)	127 (14.8)
**Increase Community Access**	**305 (35.5)**	**298 (34.7)**	**320 (37.3)**	**327 (38.1)**	**397 (46.2)**	**322 (37.5)**
Reduce socioeconomic barriers	249 (29.0)	239 (27.8)	252 (29.3)	256 (29.8)	312 (36.3)	258 (30.0)
Reduce structural barriers	157 (18.3)	151 (17.6)	174 (20.3)	177 (20.6)	230 (26.8)	179 (20.8)
**Median # of interventions implemented by facilities per month (IQR)**	4 (2-6)	4 (2-6)	4 (2-6)	4 (2-6)	5 (3-8)	4 (2-6)

© 2022 Joung RHS et al. *JAMA Network Open.*

eTable 4. Coefficients From Interrupted Time Series Models, Stratified by Disease Site

	**Time Effect (95% CI)**	***P* value**	**Level Effect (95% CI)**	***P* value**	**Trend Effect (95% CI)**	***P* value**
Overall	-13.13 (-23.07 to -3.20)	.01	101.04 (49.09 to 152.98)	<.001	36.33 (5.33 to 67.34)	.02
Breast	-15.66 (-32.48 to 1.16)	.07	164.05 (71.48 to 256.61)	.001	62.25 (-0.37 to 124.88)	.05
Colorectal	-21.01 (-29.03 to -13.0)	<.001	46.01 (24.93 to 68.0)	<.001	13.53 (6.43 to 20.63)	<.001
Lung	-0.64 (-3.03 to 1.75)	.60	9.10 (5.46 to 12.75)	<.001	1.40 (-1.08 to 3.87)	.27
Cervix	-70.28 (-163.83 to 23.28)	.14	184.58 (-3.53 to 372.69)	.05	60.32 (-32.77 to 153.40)	.20

© 2022 Joung RHS et al. *JAMA Network Open.*

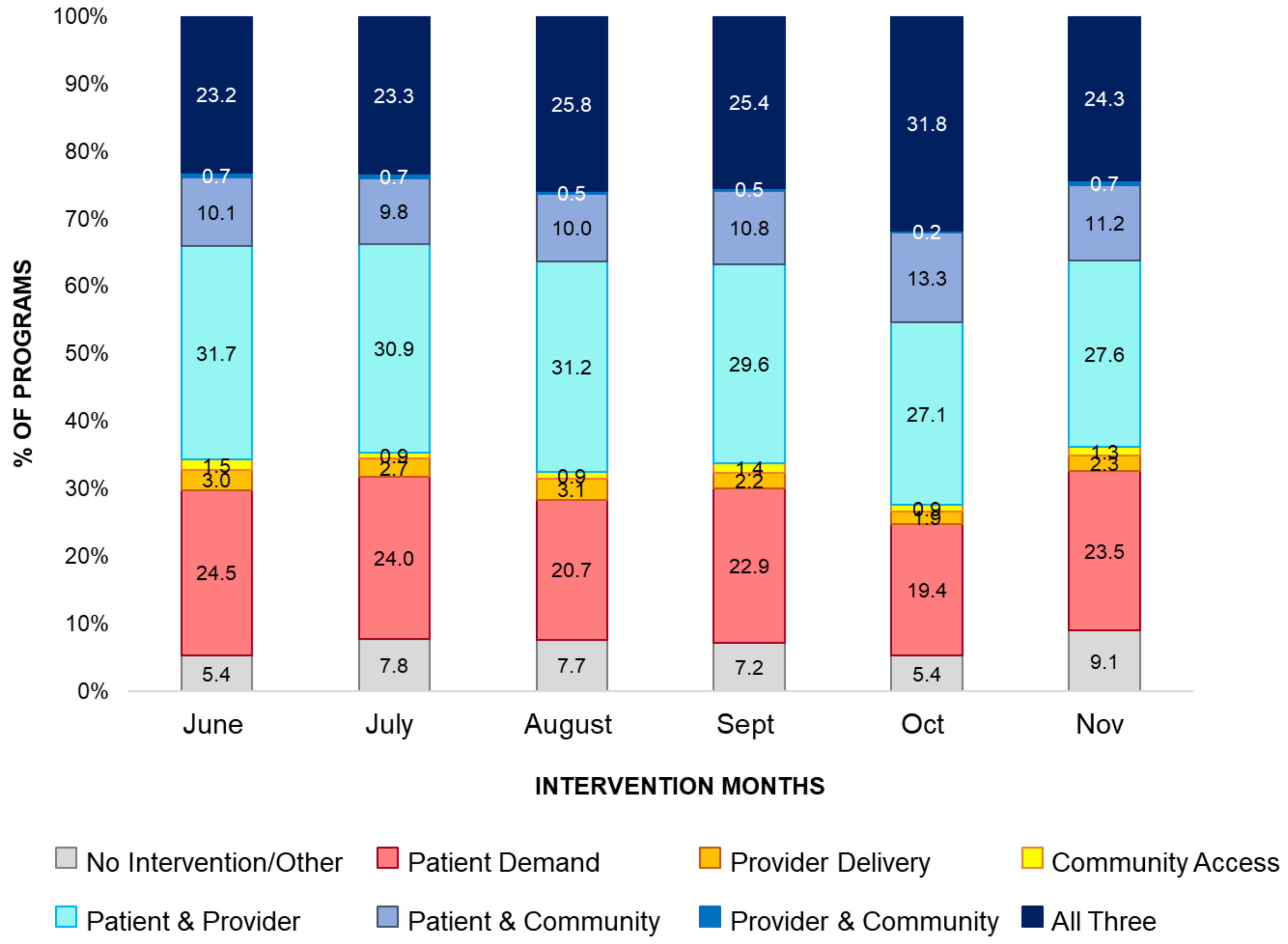

eFigure. Intervention Strategies Implemented During the Intervention Months (June to November)

© 2022 Joung RHS et al. *JAMA Network Open.*
