# Supplementary material for: Evaluation of a National Quality Improvement Collaborative for Improving Cancer Screening
Source: JAMA Netw Open. 2022 Nov 16;5(11):e2242354. doi: 10.1001/jamanetworkopen.2022.42354 (PMC9669819; doi:10.1001/jamanetworkopen.2022.42354)
Supplement: Supplement 2. — Return-to-Screening Quality Improvement Collaborative Group Members [file jamanetwopen-e2242354-s002.pdf]

Supplemental Online Content: Nonauthor Collaborators
*First name, last name, and suffix (if applicable) are required and will appear in PubMed.

*Group Name(s): Return-to-Screening Quality Improvement Collaborative

***First Name and Middle Initial(s)**	***Last Name**	***Suffix (eg, Jr, III)**	Academic Degrees	Institution	Location (city, state/province, country)	Role or Contribution, eg, chair, principal investigator	Group (if more than 1 Group listed in the byline) and/or Subgroup (eg, Steering Committee)
Ali	Abedi		MD				
Sheetal	Acharya		MD				
Karen T.	Adams		RN, BSN				
Rishi	Agarwal		MD				
Sachin	Agarwal		MD				
Rima	Ahmad		MD				
Philip	Albaneze		MD				
Kimberly	Aldis		MSN, APRN, FNP-C, OCN				
Ahkeel	Allen		MD				
Shayla	Allman		MSN, RN				
Meiling	Alsen		RN, MSNED OCN CBCN				
Mindy	Ansteth		BS, CTR, CPHQ				
Angela A.	Appiah		PhD, DNP, MSN, MPH				
Candy	Arentz		MD, MBA				
Amanda (Lori)	Arias		RT(T), MBA, ROCC				
Christine	Armetta		RN, BSN, MBA, CCRP				

Supplemental Online Content: Nonauthor Collaborators
*First name, last name, and suffix (if applicable) are required and will appear in PubMed.

***First Name and Middle Initial(s)**	***Last Name**	***Suffix (eg, Jr, III)**	Academic Degrees	Institution	Location (city, state/province, country)	Role or Contribution, eg, chair, principal investigator	Group (if more than 1 Group listed in the byline) and/or Subgroup (eg, Steering Committee)
Alicia H.	Arnold		DO				
Camelia	Arsene		MD, PhD, MHS				
Karen	Arthur		MD				
Brandon	Ashton		MD				
M Bassel	Atassi		MD				
Sameh	Attia		MBBCH				
Heidi	Bahna		MD				
Laurence	Bailen		MD				
Thelma	Baker		MSN, RN, ONC, NEA-BC				
Stefan	Balan		MD				
Amy	Balis		MD				
Anne	Balsley		RN, BSN				
Denise	Barajas		MD				
Julie L.	Barone		DO				
Brett	Batchelor		MD				
Elizabeth	Bates		MSHA, CRA				
Trevor	Bayliss		MD				
Susan	Beck		DO, MPH				
Brandon	Bennett		RN				
Ryan	Bennett		MBA				
Brandon	Bennett		RN				
Nathan	Bennett						
Jessica	Bensenhaver		MD				
Jane	Berby-Todd						
Julian	Berrocal		MD				
Danielle	Bertoni		MD				
Alison	Bevan		MD				

Supplemental Online Content: Nonauthor Collaborators
*First name, last name, and suffix (if applicable) are required and will appear in PubMed.

***First Name and Middle Initial(s)**	***Last Name**	***Suffix (eg, Jr, III)**	Academic Degrees	Institution	Location (city, state/province, country)	Role or Contribution, eg, chair, principal investigator	Group (if more than 1 Group listed in the byline) and/or Subgroup (eg, Steering Committee)
Rabia	Bhatti		MD				
Carol	Bier-Laning		MD, MBA				
Margaret M.	Blackwood		MD				
Kenneth	Blake		MD				
Joanna G.	Blankner		MD				
Joseph A.	Blansfield		MD				
Lawrence	Blaszkowsky		MD				
Brian	Blonigen		MD				
David	Bloom		MD				
Justin	Boatsman		MD				
Jonathan	Boggs		CCMA				
Richard	Bold		MD, MBA				
Deborah	Bollinger		BSN, RN, OCN				
Cheryl	Bolton						
Susan	Boolbol		MD				
Greg	Boone		MD				
Lawrence	Borges		MD, MPH				
Michael	Bouton		MD				
Carol L.	Bovest		BS				
Tara	Bowman Seitz		MD				
Carol	Boyer		RN, APN-C, AOCNS, CN-BP				
Colton	Boyle		BSN, RN				
Francisco A.	Bracho		MD				
Autumn	Bragg		MHA				
Susan	Branton		MD				
Tara M.	Breslin		MD				
Melissa N.	Brock						
Arkady	Broder		MD				

Supplemental Online Content: Nonauthor Collaborators
*First name, last name, and suffix (if applicable) are required and will appear in PubMed.

***First Name and Middle Initial(s)**	***Last Name**	***Suffix (eg, Jr, III)**	Academic Degrees	Institution	Location (city, state/province, country)	Role or Contribution, eg, chair, principal investigator	Group (if more than 1 Group listed in the byline) and/or Subgroup (eg, Steering Committee)
Christine	Brown		MS, BSN, RN, OCN				
Bradley	Brown		MD				
Holly	Brown		MSL				
Lisa M.	Brown		MD				
Richard	Brown		MD				
Laura	Bruce		MEd, BSN, RN, OCN				
Sara	Bruce		MD				
Catherine	Bruton		RHIA, CTR				
Karen	Bryant		RN, CN-BN				
William	Burak		MD				
Jessica	Burgers		MD				
Kristin	Busch		MD				
David	Caba		MD				
James	Cain		MSN, RN				
Matthew	Campbell		MD				
Cynthia	Campo		CRC				
Lynn	Canavan		MD				
Leander	Cannick		MD				
Paula	Caputo		MPA				
H. Janelle	Carr		DNP				
Christina	Casteel		MD				
Stephen	Cattaneo		MD				
Austin	Cecil		RN, ONN-CG				
Paul	Celano		MD				
Karinn	Chambers		MD				
Stella	Chambers		RT (R)(M)				

Supplemental Online Content: Nonauthor Collaborators
*First name, last name, and suffix (if applicable) are required and will appear in PubMed.

***First Name and Middle Initial(s)**	***Last Name**	***Suffix (eg, Jr, III)**	Academic Degrees	Institution	Location (city, state/province, country)	Role or Contribution, eg, chair, principal investigator	Group (if more than 1 Group listed in the byline) and/or Subgroup (eg, Steering Committee)
Janet	Chin		MD				
M Kathleen	Christian		MD				
Crystal	Chu		BSN, RN				
Allison	Church		MBA				
Jamie	Clark		BS, CTR				
Lynne P.	Clark		MD				
Marsha	Clements		RN, MSN/Ed, PhD, OCN				
Seth	Cohen		MD				
Cathy J F	Cole		NP, MPH, CHES				
Danielle	Colemire						
Julia	Compton						
Cliff P.	Connery		MD				
Delia	Constanza-Guaqueta		MD				
Stacey	Contreras		AS, CRT(M)(F)				
Alan B.	Coon		MD, PhD				
Kathleen L.	Copelen						
Javier	Corral		MD				
Rosa	Cosio		CTR				
Kimberly E.	Costas		MD				
April	Cox		DO				
John A.	Cox		MD				
Erin P.	Crane		MD				
David	Crotzer		MD				
Celeste G.	Cruz		MD				
Angelique	Cygan		RN				
Lisa	Cyphers		CTR				

Supplemental Online Content: Nonauthor Collaborators
*First name, last name, and suffix (if applicable) are required and will appear in PubMed.

***First Name and Middle Initial(s)**	***Last Name**	***Suffix (eg, Jr, III)**	Academic Degrees	Institution	Location (city, state/province, country)	Role or Contribution, eg, chair, principal investigator	Group (if more than 1 Group listed in the byline) and/or Subgroup (eg, Steering Committee)
Cheryl	Czerlanis		MD				
Paul S.	Dale		MD				
Shivang	Danak		MD				
Pragya A.	Dang		MD				
Jorge G.	Darcourt		MD				
Raj	Davuluri		MD				
Barbara	Day		MSN, APRN, FNP-BC, OCN, HPCN				
Phyllis	DeAntonio		RN, MSN				
Yamile	Der		RN MSN CNML-BC				
Nelah	DiAddezio		BSN, RN				
Leah L.	Dietrich		MD				
Edma	Diller		MPH, HSA				
Tanya	Dodge		BBA RT.R (CT)				
Gabriel	Domenech		MD				
Diana	Donovan		MSN, AOCNP				
Elizabeth	Dubil		MD				
Gary	Dunn		MD				
Lindsay	Ebling						
Bonnie	Edsall		MSN, RN				
Bogdan	Eftimie		MD				
Nemer J.	El Mouallem		MD				
Firas	Eladoumikdachi		MD				
Joy	Elliott		MD				

Supplemental Online Content: Nonauthor Collaborators
*First name, last name, and suffix (if applicable) are required and will appear in PubMed.

***First Name and Middle Initial(s)**	***Last Name**	***Suffix (eg, Jr, III)**	Academic Degrees	Institution	Location (city, state/province, country)	Role or Contribution, eg, chair, principal investigator	Group (if more than 1 Group listed in the byline) and/or Subgroup (eg, Steering Committee)
Katie	Elliott		BS, OPN-CG				
Melissa	Ellis		BSRS, CPHQ				
Kenneth	Endo		MHA				
Trisha	England		MSN, RN				
Carrie	Ennis		FACHE				
Toni	Everhart						
Amy	Evins		RN, MBA				
Matthew A.	Facktor		MD				
Celine	Fadel		DO				
Michael	Farrell						
Diane	Fawley		CTR				
Elizabeth D.	Feldman		MD				
Michael A.	Finan		MD				
Andrew	Fintel		DO				
James	Fleshman		MD				
Sara P.	Fogarty		DO				
Hiral	Fontanilla		MD				
Eric C.	Fontenot		MD				
Jon	Foran		MD				
Chaundra	Foss-Blizard		BSN, RN				
James	Frank		MD				
Julie	Franz		MD				
Lu	Freeman		CTR				
Rolf	Freter		MD, PhD				
Steven	Fried		MD				
Ryan	Gabriel		MD				
Joan	Galbraith		RPh				
Johanny	Garcia		MD				
Kim	Gardner						

Supplemental Online Content: Nonauthor Collaborators
*First name, last name, and suffix (if applicable) are required and will appear in PubMed.

***First Name and Middle Initial(s)**	***Last Name**	***Suffix (eg, Jr, III)**	Academic Degrees	Institution	Location (city, state/province, country)	Role or Contribution, eg, chair, principal investigator	Group (if more than 1 Group listed in the byline) and/or Subgroup (eg, Steering Committee)
Karen	Geary		BSW				
David J.	Gemmel		PhD				
Jessica	Gerlach		BS				
Lauren	Ghee		MD				
Maurizio	Ghisoli						
Deborah	Giannone						
Courtney	Gibbons		MHA				
Paul	Gillis		MSN				
Denis	Gilmore		MD				
Victor J	Gonzalez		MD				
Leann	Gooley		RN, BSN, OCN, CBCN				
Christine	Gorrell		MSN				
Sally	Grady		RT, BS				
David	Grew		MD				
Shaunda	Grisby		MD				
Carmen E.	Guerra		MD, MSCE				
Jacqueline	Guerriero		DO				
Kunal	Gupta		MD				
Michael	Gynn		MD				
Laura	Hafertepen		MD				
Christy	Hale		CTR, RHIT				
Ryan	Hallenbeck						
Ladonna	Hals		CTR				
Emmy L.	Hammons		BSc, AACC				
Linda B.	Haramati		MD, MS				
Natalie W.	Harper		MD, PhD				
James	Harris		MD				
Meredith A.	Harrison		MD				

Supplemental Online Content: Nonauthor Collaborators
*First name, last name, and suffix (if applicable) are required and will appear in PubMed.

***First Name and Middle Initial(s)**	***Last Name**	***Suffix (eg, Jr, III)**	Academic Degrees	Institution	Location (city, state/province, country)	Role or Contribution, eg, chair, principal investigator	Group (if more than 1 Group listed in the byline) and/or Subgroup (eg, Steering Committee)
Miranda	Harrison		RN				
John	Hassapis		MD				
Betty	Haverlock		BSN, RN, OCN, ONN-CG, CRN				
Aimee	Hawley		MD				
Theresa	Hayden		CTR, BHA				
Cherylle	Hayes		MD				
Dawn M.	Hayes		PhD, PT				
Carole	Headen		BSN, RN				
Erika	Hehnly		BSN, RN, OCN				
Sarah	Heikens		MSN MPA OCN				
Stephen	Heinzman		MD				
Mary E.	Herring		BS				
Michelle	Hill		RN, CTR				
Patricia	Hirner		MD				
Kiera	Hobbs		MSN, APRN				
Heidi	Hordyk		MBA, MSHA, RT(R), CNMT, CRA				
Neil	Horning		MD				
Donna	Howard		RT(T)				
Kan	Huang		MD, PhD				
Corilynn	Hughes						
Jenevieve	Hughes		MD				

Supplemental Online Content: Nonauthor Collaborators
*First name, last name, and suffix (if applicable) are required and will appear in PubMed.

***First Name and Middle Initial(s)**	***Last Name**	***Suffix (eg, Jr, III)**	Academic Degrees	Institution	Location (city, state/province, country)	Role or Contribution, eg, chair, principal investigator	Group (if more than 1 Group listed in the byline) and/or Subgroup (eg, Steering Committee)
Carol	Huibregtse		RN, MSN, OCN				
Nancy	Huitt		BSN, RN				
Laura	Hunsucker		RHIA				
Tina	Inverso		BSN, RN, OCN				
Tonia	Irwin		CTR, BS				
Melanie	Isbell		APRN				
Nicola	Jabbour		MD				
Mia L.	Jackson		MD				
Raymond	Jackson						
Sherly	Jacob-Perez		BS, BA, BSN, MSN, RN, CCRC				
Nazia	Jafri		MD				
Salik	Jahania		MD				
Adam	Jarrett						
Scott	Jenkinson		DO				
Richard	Johnson		MD				
Anna	Johnson						
Dianne	Johnson		MD				
Patricia	Johnson		BSN, RN, OCN, CBCN, ONN-CG				
Catherine	Johnston		RN, BSN				
Roberta	Jones		CRNP, AGN-BC, CBCN				
Susan	Jones		RN, CCM				

Supplemental Online Content: Nonauthor Collaborators
*First name, last name, and suffix (if applicable) are required and will appear in PubMed.

***First Name and Middle Initial(s)**	***Last Name**	***Suffix (eg, Jr, III)**	Academic Degrees	Institution	Location (city, state/province, country)	Role or Contribution, eg, chair, principal investigator	Group (if more than 1 Group listed in the byline) and/or Subgroup (eg, Steering Committee)
Joyce	Joseph		CTR				
Natalie E.	Joseph		MD				
Robert L.	Joyner	Jr.	PhD, RRT				
Maria	Juarez-Perez		MD				
Kimberly	Kaczmarski		MBA				
Vijaya	Kakani		MD				
Amir	Kamran		MD				
Samer	Kanaan		MD				
Tyler	Kang		MD				
Joseph	Kannarkatt		MD				
Shalini R.	Kanneganti		MD				
Belagodu	Kantharaj		MD				
Howard	Kaufman		MD, MBA				
Vickie	Keeler		RN				
Shanna	Keiser		RN, BSN				
Malissa	Kennedy		RN, PHR, CN-BN				
Iftekhar	Khan		MD				
Raza	Khan		MD				
Lesley P.	Kibel		MHA, CRA, CN-BM				
Marianne	Kiernan		RN, BSN, CN-BN, CBCN				
Brian	Kim		MD				
Adriene	Kinnaird		BS, RT(T), MBA				
Kristen L.	Kipping-Johnson		MPH				
Peggy	Kirkland		RPh, MBA				

Supplemental Online Content: Nonauthor Collaborators
*First name, last name, and suffix (if applicable) are required and will appear in PubMed.

***First Name and Middle Initial(s)**	***Last Name**	***Suffix (eg, Jr, III)**	Academic Degrees	Institution	Location (city, state/province, country)	Role or Contribution, eg, chair, principal investigator	Group (if more than 1 Group listed in the byline) and/or Subgroup (eg, Steering Committee)
Sandeep	Kirshnan		MD				
Lindy	Klaff		MD				
Robert	Kloss		MD				
Jeffrey K.	Klotz		MD				
Susan	Knight		BS, RT(R)(T), CTR				
Julie	Koch		RN, BSN, OCN, CCRP				
Paul G.	Kocheril		MD				
Dhatri	Kodali		MD				
Manpreet	Kohli		MD				
Isoken	Koko		MD, MRCP				
Amanda L.	Kong		MD, MS				
Olga	Kozyreva		MD				
Lorei A.	Kraft		BS, MT(ASCP) SH				
Stacy	Krisher		MD				
Edward J.	Kruse		DO				
Amanda	Kupstas		MD				
Mini	Kurian		RN				
Rebecca	Kwait		MD				
Cynthia	Lan		MD				
Rachelle	Lanciano		MD				
Wendy	Lannon		MS, RN, ACSM-CEP				
Sydney R.	Laster		MBA, CTR				

Supplemental Online Content: Nonauthor Collaborators
*First name, last name, and suffix (if applicable) are required and will appear in PubMed.

***First Name and Middle Initial(s)**	***Last Name**	***Suffix (eg, Jr, III)**	Academic Degrees	Institution	Location (city, state/province, country)	Role or Contribution, eg, chair, principal investigator	Group (if more than 1 Group listed in the byline) and/or Subgroup (eg, Steering Committee)
Linda R.	LaTrenta		MD				
Suzanne A.	Law						
Isabelle	Le		MD				
Nancy	Lean		MSN, MHSA, RN, NEA-BC				
Keith	Leatherbury		MD				
Jacqueline	Lee		MD				
Tyler	Leete		MD				
Mary	Legenza		MD				
Winifred K.	Leung		MD				
Jongming	Li		MD				
Anna	Likhacheva		MD				
Brian	Lingerfelt		MD				
Omar H.	Llaguna		MD				
Laura	Longo		DNP				
Melissa	Loop		RN, CN-BN				
Nicholas	Lopez		MD				
Nancy	Loporchio		BSN, RN,PHN, OCN				
Jennifer	Lowney		MD				
Carisa	Lozoraitis		RN, MSN, CPHQ				
Kit Y.	Lu		MD				
Deborah	Lue		MD				
Richard	Lush		PhD				
David T.	Luyimbazi		MD				

Supplemental Online Content: Nonauthor Collaborators
*First name, last name, and suffix (if applicable) are required and will appear in PubMed.

***First Name and Middle Initial(s)**	***Last Name**	***Suffix (eg, Jr, III)**	Academic Degrees	Institution	Location (city, state/province, country)	Role or Contribution, eg, chair, principal investigator	Group (if more than 1 Group listed in the byline) and/or Subgroup (eg, Steering Committee)
Debra	Lyons		MSN, RN, APRN, ACNP-BC				
Francisco I.	Macedo		MD				
Michelle	Machenzie		RN, OCN, CCRC				
David	Mack		MD				
Julie	Mack		MD				
Jessica	MacVicar		MD				
Kathleen L.	Mah		MD				
Kathryn	Mahoney						
Neeharika S.	Makani		MD				
Della	Makower		MD				
Salman	Malad		MD, PhD				
Cynthia	Maldonado		RN				
Farhana	Malik		MD				
Angie	Malone		DNP, APRN, ACNS-BC, OCN				
Suparna	Mantha						
Kristina	Marczak						
Nathaniel	Margolis		MD				
Kathleen	Mashanic						
Jill	Mathison		RRT, CPHQ, MBA, HCM				
Tiffany	Mauzy		BAN, RN, OCN				
Leslie	Maxwell		MBA				

Supplemental Online Content: Nonauthor Collaborators
*First name, last name, and suffix (if applicable) are required and will appear in PubMed.

*First Name and Middle Initial(s)	*Last Name	*Suffix (eg, Jr, III)	Academic Degrees	Institution	Location (city, state/province, country)	Role or Contribution, eg, chair, principal investigator	Group (if more than 1 Group listed in the byline) and/or Subgroup (eg, Steering Committee)
Jennifer	McAllaster		MD				
Caitlin	McCarthy		MD				
Jason	McClune		MD				
Jennifer K.	McColgan		PA-C, MPH				
Teresa	McCusker		BS				
Dana	McDaniel		DNP				
Lynette	McFayden		MSN				
Randall	McGivney		DO				
Kerri	McGovern		MD				
Hannah	McKeever						
Susan	McManus		MD				
Kate	McNair		PhD, WHNP-BC, RN				
Heather	McNeil						
Jennifer	McRae						
Consuelito A.	Medrano		MD				
Pallav	Mehta		MD				
Tricia	Merrigan		MD				
Eva	Meyers		PhD, NP				
Suzanne	Mickey		BS				
Jackie	Miller		MSN, RN, OCN				
Luke	Miller		MD, MS				
Stephanie	Miller		MD				
Tara	Mink		MBA, BSN, FACHE				
Jodi	Miranda		RN, BSN, BS, OCN				

Supplemental Online Content: Nonauthor Collaborators
*First name, last name, and suffix (if applicable) are required and will appear in PubMed.

***First Name and Middle Initial(s)**	***Last Name**	***Suffix (eg, Jr, III)**	Academic Degrees	Institution	Location (city, state/province, country)	Role or Contribution, eg, chair, principal investigator	Group (if more than 1 Group listed in the byline) and/or Subgroup (eg, Steering Committee)
Mary M.	Mitchell		MD				
Julie	Monroe		MD				
Joyce	Moore		MD				
Garnetta	Morin-Ducote		MD				
Troy	Moritz		DO				
Paul T.	Morris		MD				
Sumit	Mukherjee		MD, MPH, MS				
Sean D.	Mullally		MD				
Margaret	Mulligan		MD				
David A.	Mullins		MD				
Mary	Mullins		MSN				
Ivonne	Munoz		BSN, RN				
Jeremy J.	Murdock		MD				
Stacie N.	Myers		BS				
Sindhu	Nair		MD				
Kiran	Nandamuri		MD				
Joanne	Nicholls		EdD				
Donna	Nick		MSHI, CTR				
Andrew	Nish		MD				
Andrew	Nish		MD				
Andrew	Nowell		MD				
Kerri	Nowell		MD				
Lynn M.	O'Connor		MD, MPH				
Judy	O'Neill		RT(R)(T)(M)				
Moira	O'Riordan		MD				
Roselyn	Ogunkunle		PhD, MSN, RN, OCN				

*First name, last name, and suffix (if applicable) are required and will appear in PubMed.

***First Name and Middle Initial(s)**	***Last Name**	***Suffix (eg, Jr, III)**	Academic Degrees	Institution	Location (city, state/province, country)	Role or Contribution, eg, chair, principal investigator	Group (if more than 1 Group listed in the byline) and/or Subgroup (eg, Steering Committee)
Margo M.	Opsasnick		MBA				
Cindy A.	Ortiz		MBA				
Rebecca	Osgood		MD				
Jacqueline	Oxenberg		DO				
Brandi R.	Page		MD				
Jacquelyn A.V.	Palmer		MD				
Dhaval K	Parikh		MD				
David J.	Park		MD				
Jessica	Partin		MD				
Chelsea	Passwater		DNP, RN, AGCNS-BC, OCN				
Mita	Patel		MD				
Jilma	Patrick		MD				
Shauntee	Patterson						
Laura	Pearson		MD				
Susan	Peiffer						
Elizabeth	Peralta		MD				
Mary	Peterson		RN, BSN				
Benjamin	Pettus		MD, PhD				
John	Pezzulo		DO				
Megan	Pfarr		PT, DPT				
Connie	Phelps		RN, BSN				
Lacy	Phillips		MSN, RN				
Matthew	Pick						
James	Piephoff		MD				
Jodee	Pinkston		RHIT, CTR				
Jessica	Piper		MSW				
Stanley B.	Pollak		MD				
Robert	Ponec		MD				
Muhammad J.	Popalzai		MD				

*First name, last name, and suffix (if applicable) are required and will appear in PubMed.

***First Name and Middle Initial(s)**	***Last Name**	***Suffix (eg, Jr, III)**	Academic Degrees	Institution	Location (city, state/province, country)	Role or Contribution, eg, chair, principal investigator	Group (if more than 1 Group listed in the byline) and/or Subgroup (eg, Steering Committee)
Susan E.	Pories		MD				
Robert	Prieto		MD				
Kerry	Proctor		MD				
Karla	Provost						
Debra	Prow		MD				
Matthew	Puc		MD				
Mary	Purdon						
Brian P.	Quaranta		MD, MA				
Sean	Quinlan-Davidson		MD				
Alfredo	Rabines		DO				
Rakhshanda L.	Rahman		MD				
Rashmi	Ramasubbaiah		MD				
Maricarmen	Ramírez-Solá		MPHE, CN-BM				
David	Randolph	II	MD				
Elizabeth R.	Raskin		MD				
Linda	Rasmussen		MS, RN, CTR, OCN				
Nicole	Rasmussen						
Ronald	Rasmussen		MD				
Clinta C.	Reed		PhD, RN, CNL-BC				
Shellee	Reese		RN, MSN, OCN, NP-C				
Shawn M.	Regis		PhD				
James	Reilly		MD				
Richard	Reitherman		MD, PhD				
Curtis	Reneau						
Connie	Renfroe		RN				

Supplemental Online Content: Nonauthor Collaborators
*First name, last name, and suffix (if applicable) are required and will appear in PubMed.

***First Name and Middle Initial(s)**	***Last Name**	***Suffix (eg, Jr, III)**	Academic Degrees	Institution	Location (city, state/province, country)	Role or Contribution, eg, chair, principal investigator	Group (if more than 1 Group listed in the byline) and/or Subgroup (eg, Steering Committee)
Carol	Renn		CTR				
Chantal	Reyna		MD				
Kimberly	Rieger-Christ		PhD				
Rochelle	Ringer		MD				
Tina	Rizack		MD, MPH				
Uzma	Rizwan		CCS, CTR				
William R.	Robinson		MD				
Evelyn	Robles-Rodriguez		DNP, APN, AOCN				
Ethan S.	Rogers		MD				
Melissa	Ronk		RN				
George	Rossidis		MD				
Marc A.	Rovito		MD				
Abhik	Roy		MD				
Lisa	Roybal		NP				
George	Ruggiero		DO				
Rachel	Ruskin		MD				
Jessica	Ryan		MD				
Kush	Sachdeva		MD				
Anne	Saffie		CTR				
Noura	Sall		MS, CCRC				
Dana	Salters						
Juan F.	Sanchez		MD, FCCP				
Joy	Sandborn						
Michael D.	Sarap		MD				
Michael	Sarap		MD				
Jennifer	Sasaki		MD				
Jennifer	Sasaki		MD				
John M.	Schallenkamp		MD				
Lawrence E.	Schilder		DO				
Kimberly W.	Schlesinger		MD				

Supplemental Online Content: Nonauthor Collaborators
*First name, last name, and suffix (if applicable) are required and will appear in PubMed.

*First Name and Middle Initial(s)	*Last Name	*Suffix (eg, Jr, III)	Academic Degrees	Institution	Location (city, state/province, country)	Role or Contribution, eg, chair, principal investigator	Group (if more than 1 Group listed in the byline) and/or Subgroup (eg, Steering Committee)
Jackie	Scholl		RN				
Michael	Scoppetuolo		MD				
Jeanie	Scott		CTR				
Will	Sexton						
Anna	Seydel		MD				
Mazin	Shackour						
Apurva	Shah		MD				
Minal	Shah		MD				
Suhail	Sharif		MD				
Camilla M.	Shaw		BSN, RN, CN-BN				
Andrew	Shehata		DO				
Charles H	Shelton	III	MD				
Reve	Shields		MD				
Sabrina	Shilad		MD				
Eugene	Shively		MD				
Michelle	Shriner		RN, MSN, OCN, OBCN				
Joel R.	Siebentritt						
Shyamali	Singhal		MD, PhD				
Shreya	Sinha						
Suman	Sinha		MD				
Jaime	Slotkin		DO				
Kaylene	Smith		BSM, RN, OCN				
Sarah	Smith		MSN, RN, OCN, CBCN				

Supplemental Online Content: Nonauthor Collaborators
*First name, last name, and suffix (if applicable) are required and will appear in PubMed.

***First Name and Middle Initial(s)**	***Last Name**	***Suffix (eg, Jr, III)**	Academic Degrees	Institution	Location (city, state/province, country)	Role or Contribution, eg, chair, principal investigator	Group (if more than 1 Group listed in the byline) and/or Subgroup (eg, Steering Committee)
Stephanie	Smith-Marrone		MD				
Lawrence D.	Sobel		MD				
Anna	Sobolewski		MD				
Robin	Sobolewski		MD				
Naveenraj	Solomon		MD				
Tonya C.	Songy		RHIA, CTR, CPC				
Fran	Sonnier		CTR				
Leslie	Sorenson		CCRP				
Juan C.	Sorto		MD				
Will	Souza						
David M.	Spector		MD, PhD				
Zachary	Spigelman		MD				
Barbara	Steitz		LPN				
Clifford	Stephens		DNP, AGACNP-BC				
Sheelagh	Stewart		MPH, BSN, OCN				
Gretchen	Stipec		MD				
Brittany	Stith		RN, BSN, OCN, CCRP				
Holly	Street						
Janakiraman	Subramanian		MD				
Maria	Sumrall		MSN, RN				
Mark	Sundermeyer		MD				
Ali	Syed		MD				
Michael	Szwerc		MD				
Laura	Tanner		LCSW, OSW-C				

Supplemental Online Content: Nonauthor Collaborators
*First name, last name, and suffix (if applicable) are required and will appear in PubMed.

***First Name and Middle Initial(s)**	***Last Name**	***Suffix (eg, Jr, III)**	Academic Degrees	Institution	Location (city, state/province, country)	Role or Contribution, eg, chair, principal investigator	Group (if more than 1 Group listed in the byline) and/or Subgroup (eg, Steering Committee)
Tracey	Tatum		RN, MSN, FNP				
Roxana	Taveira		MHSA				
Michael	Taylor		MD				
Julia	Taylor		BA				
Christine B.	Teal		MD				
Christina	Tello-Skjerseth		MD				
Kenneth	Terhaar		MD				
Margaret	Terhar		MD				
Ravneet	Thind		MD				
Kendra L.	Thomas		BSN, RN				
Nancy	Thompson		MA, RN, OCN				
Carey C.	Thomson		MD, MPH				
Brandon	Tieu		MD				
Madhavi	Toke		MD				
Megumi	Tomita		MD				
Kenneth	Tomkovich		MD				
Elisa	Tong		MD				
Peter	Tothy		MD				
Maria	Tran		MPH, CTR				
Kay	Travis-Soper						
Gaurav	Trikha		MBBS				
Guarev	Trikha		MBBS				
Raj	Vasnani		MD				
Nirmal	Veeramachaneni		MD				
Annabelle	Veerapaneni		MD				
Deanna	Ventura-Cullen		MPH				
Dee	Vester		RN, BSN				
Julia	Vinsky		MHA				

Supplemental Online Content: Nonauthor Collaborators
*First name, last name, and suffix (if applicable) are required and will appear in PubMed.

***First Name and Middle Initial(s)**	***Last Name**	***Suffix (eg, Jr, III)**	Academic Degrees	Institution	Location (city, state/province, country)	Role or Contribution, eg, chair, principal investigator	Group (if more than 1 Group listed in the byline) and/or Subgroup (eg, Steering Committee)
Anthony	Visioni		MD				
Lindsay	Vlaminck		MS				
Akhil	Wadhera		MD				
Jamie	Wagner		DO				
Brandy	Waits		BS, RN				
Sharon	Walenga		MBA, RT(R)(M)				
Michael	Walker		MD				
Lucy B.	Wallace		MD				
James A.	Wallace		MD				
Marie	Ward		MD, MPH				
Kirsten	Warmington						
Richard	Wasley		MD				
Kimberly L.	Watson		AAS, CTR				
Wendy	Watson		RN				
Jonathan	Waxman		MD				
Robert	Weathersby		RN, BSN				
Elizabeth A.	Weaver		MD				
Mary E.	Weber		MS, CTR				
Christine	Weiselberg		DNP, FNP-BC				
Cheryl	Wesen		MD				
Ann	Wexler		MD				
Darlene	Weyer		DO				
Paul	White		MD				
Christina	White		MBA				
Karolina	Whitefield		BS				
Reagan	Wilkinson		MHA				
James C.	Willey		MD				
Verneetta	Williams		MD				
Jason P	Wilson		MD, MBA				

Supplemental Online Content: Nonauthor Collaborators
*First name, last name, and suffix (if applicable) are required and will appear in PubMed.

***First Name and Middle Initial(s)**	***Last Name**	***Suffix (eg, Jr, III)**	Academic Degrees	Institution	Location (city, state/province, country)	Role or Contribution, eg, chair, principal investigator	Group (if more than 1 Group listed in the byline) and/or Subgroup (eg, Steering Committee)
Virginia M.	Witt		MD				
Howard	Wold		MD				
Deb	Woodford		BS, RN				
Scott	Woomer		MD				
Justin W.	Wray		MD, PhD				
Karen	Yeh		MD				
Kahyun	Yoon-Flannery		DO, MPH				
Richard	Zera		MD, PhD				
Michael	Zlomke		MD				
Kirby	Zummo		MBA				
Karen	Zwicky		MS, CCRC				
